# Bis­(2-amino-6-methyl­pyridinium) tetra­bromido­cuprate(II)

**DOI:** 10.1107/S1600536808010647

**Published:** 2008-04-23

**Authors:** Rawhi H. Al-Far, Basem Fares Ali, Salim F. Haddad

**Affiliations:** a Faculty of Information Technology and Science, Al-Balqa’a Applied University, Salt, Jordan; b Department of Chemistry, Al al-Bayt University, Mafraq 25113, Jordan; c Department of Chemistry, The University of Jordan, Amman, Jordan

## Abstract

In the crystal structure of the title compound, (C_6_H_9_N_2_)_2_[CuBr_4_], the geometry around the Cu atom is inter­mediate between tetra­hedral (*T_d_*) and square planar (*D_4h_*). Each [CuBr_4_]^2−^ anion is connected non-symmetrically to four surrounding cations through N—H⋯*X* (pyridine and amine proton) hydrogen bonds, forming chains of the ladder-type running parallel to the crystallographic *b* axis. These layers are further connected by means of offset face-to-face inter­actions (parallel to the *a* axis), giving a three-dimensional network. Cation π–π stacking [centroid separations of 3.69 (9) and 3.71 (1) Å] and Br⋯aryl inter­actions [3.72 (2) and 4.04 (6) Å] are present in the crystal structure. There are no inter­molecular Br⋯Br inter­actions.

## Related literature

For related literature, see: Al-Far & Ali (2007*a* ▶,*b* ▶); Ali & Al-Far (2007 ▶, 2008 ▶); Allen *et al.* (1987 ▶, 1997 ▶); Desiraju & Steiner (1999 ▶); Dolling *et al.* (2001 ▶); Haddad *et al.* (2006 ▶); Hunter (1994 ▶); Panunto *et al.* (1987 ▶); Raithby *et al.* (2000 ▶); Robinson *et al.* (2000 ▶); Luque *et al.* (2001 ▶).
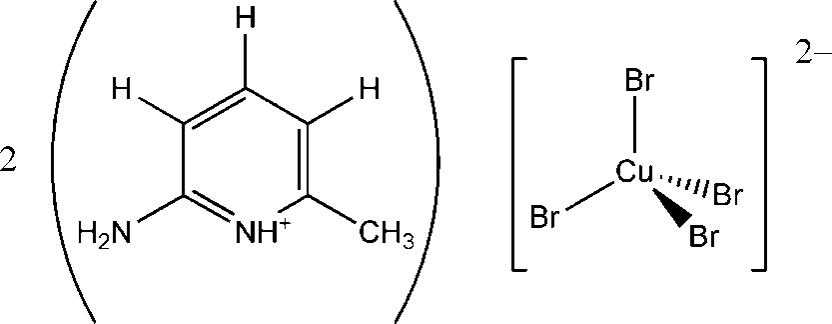


## Experimental

### 

#### Crystal data

(C_6_H_9_N_2_)_2_[CuBr_4_]
*M* *_r_* = 601.45
Triclinic, 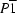

*a* = 7.9238 (9) Å
*b* = 8.2521 (11) Å
*c* = 15.2916 (18) Å
α = 78.472 (11)°
β = 82.839 (10)°
γ = 89.947 (14)°
*V* = 971.8 (2) Å^3^
*Z* = 2
Mo *K*α radiation
μ = 9.35 mm^−1^
*T* = 293 (2) K
0.20 × 0.15 × 0.10 mm

#### Data collection

Bruker P4 diffractometer
Absorption correction: ψ scan (*XSCANS*; Bruker, 1996 ▶)*T*_min_ = 0.199, *T*_max_ = 0.392
4381 measured reflections
3567 independent reflections
2018 reflections with *I* > 2σ(*I*)
*R*_int_ = 0.053

#### Refinement

*R*[*F* ^2^ > 2σ(*F* ^2^)] = 0.058
*wR*(*F* ^2^) = 0.153
*S* = 1.00
3567 reflections
190 parameters
H-atom parameters constrained
Δρ_max_ = 0.57 e Å^−3^
Δρ_min_ = −0.65 e Å^−3^

### 

Data collection: *XSCANS* (Bruker, 1996 ▶); cell refinement: *XSCANS*; data reduction: *SHELXTL* (Sheldrick, 2008 ▶); program(s) used to solve structure: *SHELXS97* (Sheldrick, 2008 ▶); program(s) used to refine structure: *SHELXL97* (Sheldrick, 2008 ▶); molecular graphics: *SHELXTL*; software used to prepare material for publication: *SHELXTL*.

## Supplementary Material

Crystal structure: contains datablocks I, global. DOI: 10.1107/S1600536808010647/at2561sup1.cif

Structure factors: contains datablocks I. DOI: 10.1107/S1600536808010647/at2561Isup2.hkl

Additional supplementary materials:  crystallographic information; 3D view; checkCIF report

## Figures and Tables

**Table:** 

Br1—Cu1	2.3848 (14)
Cu1—Br2	2.3575 (16)
Cu1—Br4	2.3713 (14)
Cu1—Br3	2.3765 (16)

**Table:** 

Br2—Cu1—Br4	101.27 (6)
Br2—Cu1—Br3	132.23 (7)
Br4—Cu1—Br3	100.99 (6)
Br2—Cu1—Br1	98.36 (6)
Br4—Cu1—Br1	129.74 (7)
Br3—Cu1—Br1	98.93 (6)

**Table 2:** Hydrogen-bond geometry (Å, °)

*D*—H⋯*A*	*D*—H	H⋯*A*	*D*⋯*A*	*D*—H⋯*A*
N1—H1⋯Br3^i^	0.86	2.47	3.324 (7)	172
N8—H8⋯Br1	0.86	2.52	3.367 (8)	170
N9—H9*B*⋯Br4^ii^	0.86	2.64	3.487 (10)	168
N2—H2*B*⋯Br2^iii^	0.86	2.73	3.547 (9)	158

Symmetry codes: (i) 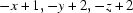; (ii) 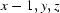; (iii) 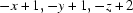.

**Fig. 1.**
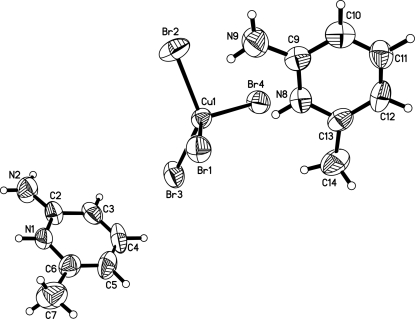
A view of the asymmetric unit of (I), with the atom-numbering scheme. Displacement ellipsoids are drawn at the 50% probability level.

**Fig. 2.**
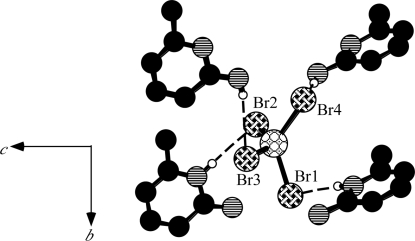
Anion···cation intermolecular interactions between one anion and four surrounding cations. N—H···Br—Cu intermolecular interactions are shown as dashed lines. Hydrogen atoms not involved in hydrogen bonding omitted for clarity.

**Fig. 3.**
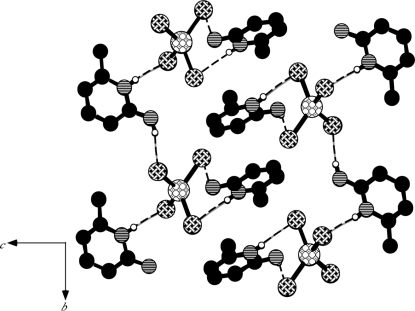
A packing diagram of (I), shows chains of the ladder type run approximately parallel to the crystallographic b-axis. Hydrogen atoms omitted for clarity.

## supplementary crystallographic information

### Comment

Non-covalent interactions play an important role in organizing structural units in both natural and artificial systems. They exercise important effects on the organization and properties of many materials in areas such as biology (Hunter 1994; Desiraju & Steiner 1999), crystal engineering (see for example: Allen *et al.*,1997; Dolling *et al.*, 2001) and material science (Panunto *et al.*, 1987; Robinson *et al.*, 2000). The interactions governing the crystal organization are expected to affect the packing and then the specific properties of solids. In connection with ongoing studies (Ali & Al-Far, 2008; Ali & Al-Far, 2007; Al-Far & Ali, 2007a,b) of the structural aspects of halo-metal anion salts, we herein report the crystal structure of title compound (I) along with its crystal supramolecularity.

The asymmetric unit in (I) contains one anion and two cations (Fig. 1). The Cu—Br distances are similar, but Cu—Br2 that is engaged in longest hydrogen bonding is shorter than the others (Table 2). The Cu—Br bond distances fall in the range of bond distances reported previously for compounds containing Cu—Br anions (Luque *et al.*, 2001; Raithby *et al.*, 2000; Haddad *et al.*, 2006). The bond angles are present in two distinguished sets. The first contains four angles in the range 98.36 (6) - 101.27 (6)° which are much lower than the other set which contains two angles 129.74 (7) and 132.23 (7)°. Accordingly the geometry of CuBr_4_^2-^ anion is an intermediate between regular tetrahedral (*T_d_*) and square planar (*D_4h_*) (Table 2).

The cation bond lengths and angles are within expected range (Allen *et al.* 1987), with the cations (type A contains N1 and type B contains N8) being of course planar.

In the structure (Fig. 2), each anion is connected nonsymmetrically to four cations interacting *via* N—H···Br and HN—H···Br hydrogen bonding, Table 3, forming chains of the ladder type run approximately parallel to the crystallographic *b*-axis. The cations type A represnt the rungs while both cations type B and anions represent the rails of a ladder (Fig. 3).

There are no Br···Br interactions were observed (shortest being 4.6651 (17) Å). Cations π···π stacking (in *a*-ditrection) is observed, with significant ones being X1A···X1A [2 - *x*, 2 - *y*, 2 - *z*] and X1B···X1B [- *x*, 1 - *y*, 1 - *z*] of 3.69 (9) and 3.71 (1), respectively. Also Br···aryl interactions present by the unusually short Br(1) [-*x*, 2 - *y*, 1 - *z*]···X1B contact of 3.72 (2) Å and the longer Br(3)···X1A of 4.04 (6) Å contact.

### Experimental

To a warm solution of 2-amino-6-methylpyridine (2 mmol) dissolved in 10 ml absolute ethanol acidified with 3 ml 60% HBr, CuBr_2_ (1 mmol) dissolved in 10 ml absolute ethanol was added. The resulting solution was then treated with 2 ml of Br_2 (_*_l_*_)_. The mixture was refluxed for 2 h. The mixture was then allowed to stand and evaporate slowly at room temperature. In two days time, block blue crystals were formed and filtered (yield, 86.5%). A single-crystal suitable for diffraction measurements were chosen and used for data collection.

### Refinement

H atoms bound to carbon and nitrogen were placed at idealized positions [C—H = 0.93 and 0.96 Å and N—H = 0.86 Å] and allowed to ride on their parent atoms with *U_iso_* fixed at 1.2 or 1.5 *U_eq_*(C,*N*).

### Crystal data

**Table:**

(C_6_H_9_N_2_)_2_[CuBr_4_]	*Z* = 2
*M**_r_* = 601.45	*F*_000_ = 574
Triclinic, *P*1	*D*_x_ = 2.056 Mg m^−^^3^
Hall symbol: -P 1	Mo *K*α radiation λ = 0.71073 Å
*a* = 7.9238 (9) Å	Cell parameters from 298 reflections
*b* = 8.2521 (11) Å	θ = 2.2–27.5º
*c* = 15.2916 (18) Å	µ = 9.35 mm^−^^1^
α = 78.472 (11)º	*T* = 293 (2) K
β = 82.839 (10)º	Block, blue
γ = 89.947 (14)º	0.20 × 0.15 × 0.10 mm
*V* = 971.8 (2) Å^3^	

### Data collection

**Table:**

Bruker P4 diffractometer	3567 independent reflections
Radiation source: fine-focus sealed tube	2018 reflections with *I* > 2σ(*I*)
Monochromator: graphite	*R*_int_ = 0.053
Detector resolution: 3 pixels mm^-1^	θ_max_ = 25.5º
*T* = 293(2) K	θ_min_ = 2.5º
ω scans	*h* = −9→1
Absorption correction: ψ scan(PROGRAM; REF (YEAR)	*k* = −9→9
*T*_min_ = 0.199, *T*_max_ = 0.392	*l* = −18→18
4381 measured reflections	

### Refinement

**Table:**

Refinement on *F*^2^	Secondary atom site location: difference Fourier map
Least-squares matrix: full	Hydrogen site location: inferred from neighbouring sites
*R*[*F*^2^ > 2σ(*F*^2^)] = 0.058	H-atom parameters constrained
*wR*(*F*^2^) = 0.153	*w* = 1/[σ^2^(*F*_o_^2^) + (0.0724*P*)^2^] where *P* = (*F*_o_^2^ + 2*F*_c_^2^)/3
*S* = 1.00	(Δ/σ)_max_ < 0.001
3567 reflections	Δρ_max_ = 0.57 e Å^−^^3^
190 parameters	Δρ_min_ = −0.65 e Å^−^^3^
Primary atom site location: structure-invariant direct methods	Extinction correction: none

### Special details

**Table:**

Geometry. All e.s.d.'s (except the e.s.d. in the dihedral angle between two l.s. planes) are estimated using the full covariance matrix. The cell e.s.d.'s are taken into account individually in the estimation of e.s.d.'s in distances, angles and torsion angles; correlations between e.s.d.'s in cell parameters are only used when they are defined by crystal symmetry. An approximate (isotropic) treatment of cell e.s.d.'s is used for estimating e.s.d.'s involving l.s. planes.
Refinement. Refinement of *F*^2^ against ALL reflections. The weighted *R*-factor *wR* and goodness of fit *S* are based on *F*^2^, conventional *R*-factors *R* are based on *F*, with *F* set to zero for negative *F*^2^. The threshold expression of *F*^2^ > σ(*F*^2^) is used only for calculating *R*-factors(gt) *etc*. and is not relevant to the choice of reflections for refinement. *R*-factors based on *F*^2^ are statistically about twice as large as those based on *F*, and *R*- factors based on ALL data will be even larger.

### Fractional atomic coordinates and isotropic or equivalent isotropic displacement parameters (Å^2^)

**Table:**

	*x*	*y*	*z*	*U*_iso_*/*U*_eq_	
Br1	0.18757 (14)	0.94944 (11)	0.69574 (7)	0.0597 (3)	
Cu1	0.32354 (14)	0.69646 (13)	0.74711 (8)	0.0442 (3)	
N1	0.6876 (10)	1.0718 (9)	1.0416 (6)	0.052 (2)	
H1	0.6292	1.1120	1.0832	0.062*	
Br2	0.08231 (15)	0.54289 (13)	0.82710 (9)	0.0745 (4)	
N2	0.6864 (13)	0.8282 (10)	1.1440 (6)	0.074 (3)	
H2A	0.6268	0.8750	1.1825	0.089*	
H2B	0.7143	0.7266	1.1591	0.089*	
C2	0.7347 (13)	0.9116 (12)	1.0624 (7)	0.052 (3)	
Br3	0.56567 (13)	0.80573 (15)	0.79499 (8)	0.0679 (4)	
C3	0.8257 (13)	0.8462 (13)	0.9938 (8)	0.060 (3)	
H3	0.8594	0.7368	1.0049	0.072*	
Br4	0.45192 (14)	0.50445 (13)	0.66438 (8)	0.0650 (4)	
C4	0.8653 (14)	0.9439 (17)	0.9100 (8)	0.073 (4)	
H4	0.9239	0.9006	0.8636	0.087*	
C5	0.8171 (15)	1.1093 (15)	0.8947 (7)	0.070 (3)	
H5	0.8487	1.1761	0.8384	0.084*	
C6	0.7270 (14)	1.1742 (12)	0.9585 (7)	0.058 (3)	
C7	0.6683 (17)	1.3493 (13)	0.9503 (9)	0.089 (4)	
H7C	0.7028	1.4097	0.8902	0.134*	
H7B	0.7180	1.4007	0.9923	0.134*	
H7A	0.5465	1.3491	0.9630	0.134*	
N8	0.0896 (10)	0.7845 (9)	0.5235 (5)	0.049 (2)	
H8	0.1279	0.8242	0.5653	0.059*	
C9	−0.0722 (12)	0.7230 (11)	0.5402 (7)	0.049 (2)	
N9	−0.1600 (12)	0.7226 (11)	0.6204 (7)	0.080 (3)	
H9A	−0.1137	0.7608	0.6603	0.095*	
H9B	−0.2629	0.6841	0.6322	0.095*	
C10	−0.1360 (13)	0.6650 (11)	0.4711 (8)	0.057 (3)	
H10	−0.2473	0.6239	0.4788	0.068*	
C11	−0.0343 (14)	0.6690 (12)	0.3923 (8)	0.057 (3)	
H11	−0.0771	0.6302	0.3461	0.069*	
C12	0.1301 (15)	0.7289 (12)	0.3794 (7)	0.062 (3)	
H12	0.1968	0.7290	0.3249	0.074*	
C13	0.1985 (13)	0.7888 (11)	0.4451 (7)	0.052 (3)	
C14	0.3706 (14)	0.8601 (14)	0.4410 (8)	0.075 (3)	
H14A	0.3823	0.8923	0.4969	0.112*	
H14B	0.3878	0.9553	0.3929	0.112*	
H14C	0.4538	0.7792	0.4306	0.112*	

### Atomic displacement parameters (Å^2^)

**Table:**

	*U*^11^	*U*^22^	*U*^33^	*U*^12^	*U*^13^	*U*^23^
Br1	0.0758 (8)	0.0456 (6)	0.0583 (7)	0.0137 (5)	−0.0121 (6)	−0.0101 (5)
Cu1	0.0410 (7)	0.0451 (6)	0.0453 (7)	0.0029 (5)	0.0001 (5)	−0.0096 (5)
N1	0.049 (5)	0.058 (5)	0.050 (6)	−0.003 (4)	0.005 (4)	−0.023 (4)
Br2	0.0557 (7)	0.0607 (7)	0.0916 (10)	−0.0022 (5)	0.0223 (7)	0.0021 (6)
N2	0.116 (9)	0.060 (5)	0.045 (6)	0.010 (5)	−0.009 (6)	−0.003 (5)
C2	0.067 (7)	0.050 (6)	0.046 (7)	0.015 (5)	−0.013 (6)	−0.018 (5)
Br3	0.0443 (6)	0.1028 (9)	0.0670 (8)	−0.0010 (6)	−0.0037 (6)	−0.0441 (7)
C3	0.052 (7)	0.067 (7)	0.071 (9)	0.000 (5)	−0.013 (6)	−0.034 (7)
Br4	0.0553 (7)	0.0640 (7)	0.0793 (9)	0.0006 (5)	0.0101 (6)	−0.0345 (6)
C4	0.050 (7)	0.125 (11)	0.061 (9)	0.010 (7)	−0.010 (6)	−0.060 (8)
C5	0.076 (8)	0.100 (9)	0.035 (7)	0.001 (7)	−0.010 (6)	−0.013 (6)
C6	0.063 (7)	0.063 (6)	0.044 (7)	−0.002 (5)	−0.010 (6)	−0.003 (5)
C7	0.091 (10)	0.069 (8)	0.098 (11)	0.009 (7)	0.000 (8)	0.001 (7)
N8	0.050 (5)	0.055 (5)	0.045 (5)	0.008 (4)	−0.009 (4)	−0.014 (4)
C9	0.037 (6)	0.049 (5)	0.059 (7)	0.009 (4)	−0.002 (5)	−0.010 (5)
N9	0.058 (6)	0.108 (8)	0.080 (8)	−0.007 (5)	0.009 (6)	−0.048 (6)
C10	0.048 (6)	0.053 (6)	0.069 (8)	0.010 (5)	−0.020 (6)	−0.001 (6)
C11	0.057 (7)	0.070 (7)	0.049 (7)	0.007 (6)	−0.022 (6)	−0.011 (5)
C12	0.079 (9)	0.070 (7)	0.035 (6)	0.017 (6)	−0.005 (6)	−0.005 (5)
C13	0.055 (6)	0.042 (5)	0.053 (7)	0.003 (5)	0.002 (6)	0.001 (5)
C14	0.050 (7)	0.091 (8)	0.075 (9)	−0.006 (6)	0.008 (6)	−0.006 (7)

### Geometric parameters (Å, °)

**Table:**

Br1—Cu1	2.3848 (14)	C7—H7B	0.9600
Cu1—Br2	2.3575 (16)	C7—H7A	0.9600
Cu1—Br4	2.3713 (14)	N8—C9	1.355 (12)
Cu1—Br3	2.3765 (16)	N8—C13	1.382 (12)
N1—C2	1.360 (11)	N8—H8	0.8600
N1—C6	1.377 (13)	C9—N9	1.331 (13)
N1—H1	0.8600	C9—C10	1.391 (14)
N2—C2	1.310 (13)	N9—H9A	0.8600
N2—H2A	0.8600	N9—H9B	0.8600
N2—H2B	0.8600	C10—C11	1.359 (14)
C2—C3	1.396 (13)	C10—H10	0.9300
C3—C4	1.371 (16)	C11—C12	1.370 (15)
C3—H3	0.9300	C11—H11	0.9300
C4—C5	1.399 (15)	C12—C13	1.371 (14)
C4—H4	0.9300	C12—H12	0.9300
C5—C6	1.335 (14)	C13—C14	1.475 (14)
C5—H5	0.9300	C14—H14A	0.9600
C6—C7	1.504 (13)	C14—H14B	0.9600
C7—H7C	0.9600	C14—H14C	0.9600
			
Br2—Cu1—Br4	101.27 (6)	C6—C7—H7A	109.5
Br2—Cu1—Br3	132.23 (7)	H7C—C7—H7A	109.5
Br4—Cu1—Br3	100.99 (6)	H7B—C7—H7A	109.5
Br2—Cu1—Br1	98.36 (6)	C9—N8—C13	125.5 (9)
Br4—Cu1—Br1	129.74 (7)	C9—N8—H8	117.2
Br3—Cu1—Br1	98.93 (6)	C13—N8—H8	117.2
C2—N1—C6	124.6 (8)	N9—C9—N8	118.6 (10)
C2—N1—H1	117.7	N9—C9—C10	124.3 (10)
C6—N1—H1	117.7	N8—C9—C10	117.1 (9)
C2—N2—H2A	120.0	C9—N9—H9A	120.0
C2—N2—H2B	120.0	C9—N9—H9B	120.0
H2A—N2—H2B	120.0	H9A—N9—H9B	120.0
N2—C2—N1	117.9 (9)	C11—C10—C9	119.4 (10)
N2—C2—C3	124.8 (9)	C11—C10—H10	120.3
N1—C2—C3	117.3 (10)	C9—C10—H10	120.3
C4—C3—C2	119.8 (10)	C10—C11—C12	121.3 (10)
C4—C3—H3	120.1	C10—C11—H11	119.4
C2—C3—H3	120.1	C12—C11—H11	119.4
C3—C4—C5	119.5 (10)	C11—C12—C13	121.6 (10)
C3—C4—H4	120.3	C11—C12—H12	119.2
C5—C4—H4	120.3	C13—C12—H12	119.2
C6—C5—C4	122.0 (11)	C12—C13—N8	115.1 (10)
C6—C5—H5	119.0	C12—C13—C14	128.1 (11)
C4—C5—H5	119.0	N8—C13—C14	116.8 (10)
C5—C6—N1	116.8 (10)	C13—C14—H14A	109.5
C5—C6—C7	126.9 (11)	C13—C14—H14B	109.5
N1—C6—C7	116.3 (9)	H14A—C14—H14B	109.5
C6—C7—H7C	109.5	C13—C14—H14C	109.5
C6—C7—H7B	109.5	H14A—C14—H14C	109.5
H7C—C7—H7B	109.5	H14B—C14—H14C	109.5
			
C6—N1—C2—N2	−179.2 (10)	C13—N8—C9—N9	177.8 (9)
C6—N1—C2—C3	−1.6 (15)	C13—N8—C9—C10	−2.5 (13)
N2—C2—C3—C4	178.1 (11)	N9—C9—C10—C11	−179.0 (10)
N1—C2—C3—C4	0.7 (15)	N8—C9—C10—C11	1.4 (13)
C2—C3—C4—C5	1.4 (16)	C9—C10—C11—C12	0.1 (15)
C3—C4—C5—C6	−2.8 (17)	C10—C11—C12—C13	−0.6 (16)
C4—C5—C6—N1	1.9 (17)	C11—C12—C13—N8	−0.3 (14)
C4—C5—C6—C7	179.9 (11)	C11—C12—C13—C14	−178.6 (10)
C2—N1—C6—C5	0.3 (16)	C9—N8—C13—C12	2.0 (14)
C2—N1—C6—C7	−177.9 (9)	C9—N8—C13—C14	−179.5 (9)

### Hydrogen-bond geometry (Å, °)

**Table:**

*D*—H···*A*	*D*—H	H···*A*	*D*···*A*	*D*—H···*A*
N1—H1···Br3^i^	0.86	2.47	3.324 (7)	172
N8—H8···Br1	0.86	2.52	3.367 (8)	170
N9—H9B···Br4^ii^	0.86	2.64	3.487 (10)	168
N2—H2B···Br2^iii^	0.86	2.73	3.547 (9)	158

Symmetry codes: (i) −*x*+1, −*y*+2, −*z*+2; (ii) *x*−1, *y*, *z*; (iii) −*x*+1, −*y*+1, −*z*+2.
